# The calming effect of a new wearable device during the anticipation of public speech

**DOI:** 10.1038/s41598-017-02274-2

**Published:** 2017-05-23

**Authors:** Ruben T. Azevedo, Nell Bennett, Andreas Bilicki, Jack Hooper, Fotini Markopoulou, Manos Tsakiris

**Affiliations:** 1 Lab of Action & Body, Department of Psychology, Royal Holloway, University of London, Egham, TW20 0EX UK; 2 Team Turquoise Ltd, 1 Bermondsey Square, London, SE1 3UN UK

## Abstract

We assessed the calming effect of *doppel*, a wearable device that delivers heartbeat-like tactile stimulation on the wrist. We tested whether the use of *doppel* would have a calming effect on physiological arousal and subjective reports of state anxiety during the anticipation of public speech, a validated experimental task that is known to induce anxiety. Two groups of participants were tested in a single-blind design. Both groups wore the device on their wrist during the anticipation of public speech, and were given the cover story that the device was measuring blood pressure. For only one group, the device was turned on and delivered a slow heartbeat-like vibration. Participants in the *doppel* active condition displayed lower increases in skin conductance responses relative to baseline and reported lower anxiety levels compared to the control group. Therefore, the presence, as opposed to its absence, of a slow rhythm, which in the present study was instantiated as an auxiliary slow heartbeat delivered through *doppel*, had a significant calming effect on physiological arousal and subjective experience during a socially stressful situation. This finding is discussed in relation to past research on responses and entrainment to rhythms, and their effects on arousal and mood.

## Introduction

Wearable devices are becoming ubiquitous in everyday life, serving a range of functions, from measuring physical activity and a range of physiological variables to providing feedback on emotional states. Across most such instantiations of wearable technologies, the overarching aim seems to be the quantification of the self^1^. However, as recent studies suggest, the effects of such tracking devices are debatable^2^, seem to be short-lived, and consumers often stop using them after a short period of time^3^. As a result, questions about what would be a more intuitive wearable device that can assist people in their everyday life have motivated the development and design of a new wearable device by Team Turquoise Ltd (http://www.doppel.london/). The *doppel* device, rather than measuring physiological variables, aims at providing a sensory experience to help people manage the pressures of time and stress in their daily lives by modulating the users’ physiological and potentially emotional and cognitive states during a wide range of everyday life tasks. The *doppel* delivers an on-demand, discrete, user-controlled, heartbeat-like vibration applied through a wristband. In the final product, the wristband is connected via Bluetooth to a smart phone app where the user measures their resting heart rate and chooses their preferred speed and intensity of tactile stimulation in relation to their own heartrate.

The design of *doppel* was inspired by several research strands that show how humans respond to and possibly entrain to different rhythms. Entrainment is a broadly used term that, in the context of human behavior and physiology, reflects the voluntary or involuntary synchronization of our brains and bodies to the environment^4^. For example, the tempo of a song can naturally alter our breathing rate and heart rate^5^. As a real-life example, choir singers not only harmonise their voices, they can also synchronise their heartbeats^6^. Vickhoff and colleagues^6^ monitored the heart rates of singers and found that as the members sang in unison, their pulses began to speed up and slow down at the same rate. Beyond music, several studies highlight entrainment effects in responses to biological rhythms, and the heartbeat is perhaps the most ubiquitous biological rhythm in nature. The mother’s own heartbeat is a particular salient multisensory signal that accompanies the development of the foetus and, not surprisingly, has been shown to have beneficial effects for the infant’s development. For example, the perception of the maternal heartbeat in the womb provides the foetus with an important rhythmic experience^7^ that is thought to facilitate the formation of the neural basis for entrainment and synchrony skills necessary for a wide range of cognitive processes that underpin vocal, gestural, and gaze communication during mother–infant interactions^8^. Such entrainment effects to physiological rhythms are evidenced in a wide range of contexts in later life. For example, the heartbeats of a mother and baby will synchronise with one another when they interact closely^9^, and similar effects have been observed in couples^10^.

Beyond specific entrainment effects, more general, our brains and bodies also respond naturally to rhythm. In music, tempo has been consistently shown to play an important role in mood induction and physiological changes^11–14^. For example, listening to slower tempo results in lower arousal and subjective states of positive or calm emotional states^12, 15–17^, see ref. 18 for review. Conversely, fast rhythms are associated with high arousal and positive and/or arousing emotional states such as joy, excitement, surprise, fear or anger^12, 18^. Beyond the perception of tempo in music, the physiological importance of faster and slower heart rate as well as of high and low heart rate variability for physical and mental health is well-documented, as well as their role for experienced arousal and cognitive processing in general^19^. Not only high arousal is physiologically correlated with increased heart rate, and calmness with lower heart rate, as we also intuitively associate higher and lower heart rate with anxiety or high arousal and calmness, respectively. Based on the aforementioned lines of research about the importance of heartbeats as pervasive physiological signals involved in emotional regulation and social interactions, and the effects of slow rhythms on mood, *doppel* was designed to embed on the users’ body, the ubiquitous biological rhythm of a beating heart as a means of using the felt faster or slower rhythm to modulate levels of arousal and calmness. To that end, the present study was designed to test whether the use of *doppel*, as opposed to its absence, could have a calming effect on physiological arousal and subjective reports of experienced anxiety during a stressful situation.

Stressful situations are typically associated with increases in physiological arousal and negative affect. However, stressful contexts differ from one another in character. For example, a mental arithmetic challenge differs from a respiratory challenge, which both differ from a social stress challenge. Socially stressful situations may involve the threat of negative social evaluation (e.g., public speaking challenge) or may be accompanied by negative social evaluation resulting in social rejection (e.g., social exclusion manipulation). Given the significance of interoception in cognitive-affective processing^20–22^, potential changes in interoceptive signals (i.e. signals originating from visceral organs such as the heart) in stressful, negative and affective situations may influence emotional experience, emotion regulation and decision making in these contexts. In such situations, the ability to regulate one’s physiological arousal and emotions may be important for lowering anxiety and for successful performance.

A classic task that has been used in controlled psychological experiments to elicit an ecologically valid and socially stressful situation, is the anticipation of public speech task. Across a large number of studies, it has been shown that anticipation of public speaking is particularly effective at inducing social anxiety with concomitant increases in physiological arousal^23–26^. Therefore, the current study utilized the speech anticipation manipulation to examine how the use of *doppel* could enable participants to cope better with their anxiety while they were preparing to deliver a speech to a group of people. Moreover, given the intended use of the product, we were particularly interested in testing the efficacy of its use against a condition where participants were not using it. Importantly, for the purposes of this study, participants were unaware of the true function of *doppel* as we wanted to minimize expectations and potential confounds. To that end, participants were told that *doppel* was a device designed to measure their blood pressure. Two groups of participants were tested. Both groups wore the device on their wrist, but only for one group *doppel* was turned on and vibrated during the anticipation period. *Doppel* was set to vibrate with a frequency ~20% slower than the participant’s heartbeats, as measured at rest, because previous research has shown that slower rhythms tend to be associated with low arousal and subjective states of calmness/serenity, sadness or boredom^18^. Before instructions were given and throughout the anticipatory period, levels of physiological arousal were measured through continuous recordings of skin conductance and heart rate. In addition, we collected psychometric data to quantify state anxiety levels before and after the anticipation of public speech task. It was hypothesized, that the *doppel device* active group would display lower increases in physiological arousal and report lower levels of state anxiety. It should be noted that the method adopted here with *doppel* differs from biofeedback or false feedback techniques^27–29^ in which exteroceptive stimulation reflects ongoing cardiac activity and participants are encouraged to pay attention and/or regulate it. Instead, we tested whether periodic vibrotactile stimulation unrelated to participant’s ongoing heartbeats, as opposed to the absence of such stimulation, could have a positive calming effect.

## Methods

### Participants

A total of 52 (20 Male; mean age = 26.4, s.d. = 5.7) volunteers took part in the study. Twenty five participants (9 Male; mean age = 25.9, s.d. = 5.2) were assigned to the experimental group (i.e. *doppel active condition*) and 27 (11 Male; mean age = 26.8, s.d. = 6.1) to the control group (i.e. *doppel turned off* condition). Participants were randomly assigned to each group prior to study start. All participants provided written informed consent to take part in this study, which was approved by the College Ethics Committee of Royal Holloway University of London research ethics committee, and all methods were performed in accordance with the relevant guidelines and regulations of the College Ethics Committee.

### Procedure

**Figure 1:**
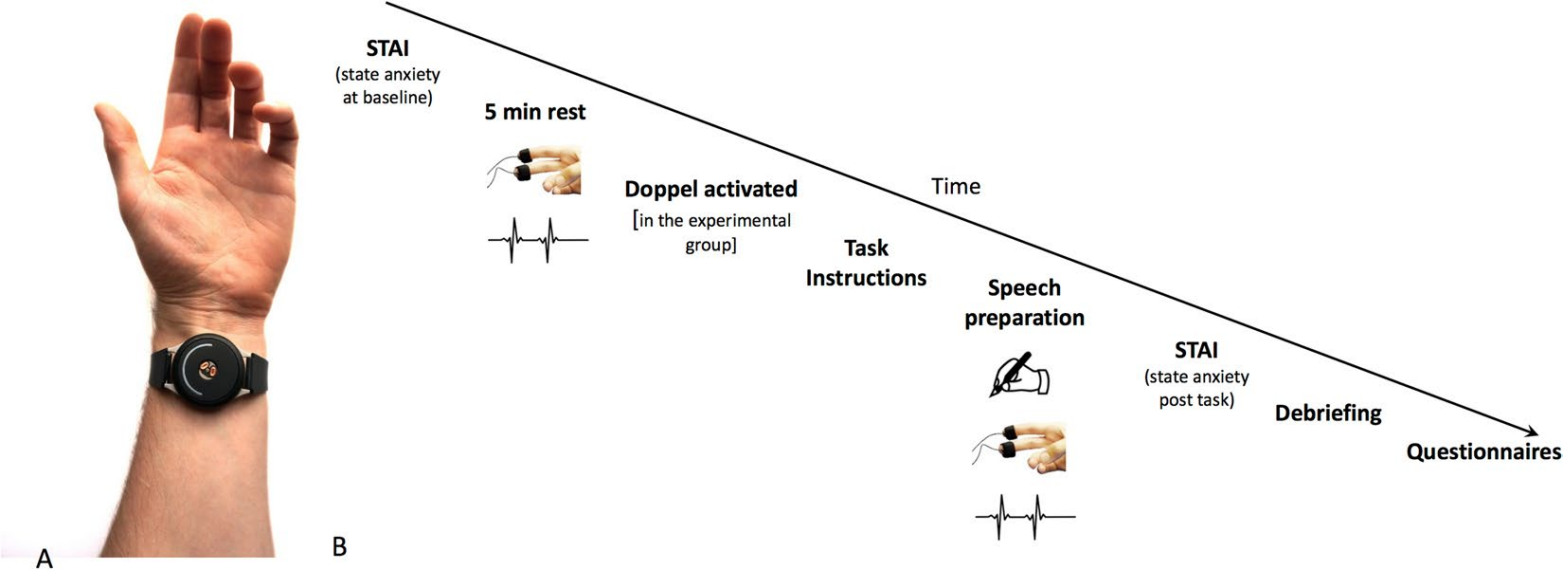
The doppel device (**A**) and the timeline of the experimental procedure (**B**).

A graphical illustration of the study’s procedure and timeline can be seen in Fig. 1. Participants were comfortably sat in an armchair, given written information about the study and asked to sign a consent form. Skin conductance Ag/AgCl electrodes (MLT117F) with 0.5%-NaCl electrode gel were attached to the phalanges of the middle and ring fingers of the participant’s non-dominant hand. The signal was recorded using a Powerlab 8/35 (https://www.adinstruments.com/), a GSR Amp unit (22 mV constant voltage at 75HZ) and LabChart (v 8.1) software with a recording range of 40 µS and a sampling rate of 1 kHz. A pulse transducer (TN1012/ST; https://www.adinstruments.com/) was also attached to the thumb of non-dominant hand to monitor cardiac activity.

The *doppel device* (see Fig. 1A) was fitted to the participant’s non-dominant wrist, in a firm but not tight position, and remained off until the period preceding task instructions. The device sits on the inside of the wrist where users expect to feel their pulse. The unit is 38 mm in diameter & 9 mm thick, held in place with a silicon strap. The device provides a double heartbeat-like rhythm tactile sensation. The main casing has a steep metal base and a plastic cover. The casing houses the electronic components: a coin vibration motor that delivers the stimulus, a low energy Bluetooth chip that communicates with an app or computer to upload new rhythms to the device, and a lithium ion battery that powers the device.

For the purposes of this study, participants were told that it was a new generation device to monitor blood pressure, and none of the participants expressed any suspicion about the purpose of the device at any point throughout the entire duration of the study. A single-blind protocol was adopted as necessary to provide the experimenter with appropriate control over *doppel* (i.e. switch off to those assigned to the control group) and to prevent participants from using cognitive strategies during the task related to the study’s goals.

At the beginning of the experiment, participants were instructed to rest for 5 minutes while sitting still, with the purpose of measuring baseline levels of physiological activity. Average heart rate and skin conductance levels during this period were taken as indices of pre-task physiological activity. After this rest period, the experimenter told participants “I am going to change the settings of the device you have on the wrist to start measuring blood pressure. You might feel some vibration. If you do, don’t worry it’s normal”. The device was then turned on in all cases but switched off after 10 seconds if the participant had been assigned to the control group. The device’s vibration frequency was individually tailored to a frequency ~20% slower (mean = 58.2, s.d. = 6.3) than the participant’s baseline HR (mean = 75.8, s.d. = 12.3), as measured during the rest period. However, upper (65 bpm) and lower (40 bpm) frequency limits were imposed to prevent vibrations to be perceived as fast or unnaturally slow, respectively. In the experimental group, the lower limit was applied to one participant (rest HR = 44.7) and the upper limit to 9 participants (mean rest HR = 88.1, s.d. = 5.5). The average absolute adjustment to lower and upper limits was of 4.4 bpm (s.d. = 3.9). Next, task instructions were provided verbally by the experimenter: “You should now prepare a speech about the use of animals for research. You may present both the pros and cons of using animals in research or give your personal point of view on this issue. You can use this pen and paper to take notes if you wish to. You have 5 minutes to prepare it. After that we will move to the room next door where you should give the speech to 3 or 4 colleagues of mine. The speech should last for 5 minutes”.

At the end of the study, to assess the success of our manipulation, participants were invited, in a semi-structured way, to share their subjective experiences during task. First they were asked “how were you feeling about giving a speech to unfamiliar people”? Then the experimenter directly enquired if participants believed they would actually give the speech. All participants, but one, were convinced they would give the speech.

### Subjective Measures

Participants were asked to complete the STAI-Y-1^30^ twice to measure self-reported anxiety levels before the delivery of task instructions and after the task completion. The STAI-Y-1 questionnaire is composed of 20 items (e.g. “I am tense”; “I feel secure”) assessing state anxiety. Each item is rated on a 4-point scale ranging from “not at all” to “very much so”. High scores reflect high anxiety levels. To control for possible differences between groups in trait apprehension about other’s evaluations, participants were asked to complete the Brief Fear of Negative Evaluation questionnaire (bFNE)^31^. The bFNE is composed of 12 items (e.g. “I am afraid others will not approve of me”) scored on a 5-point scale ranging from “not at all characteristic of me” to “extremely characteristic of me”. High scores reflect high concern about others’ evaluation. To control for possible between-group differences in participants’ general disposition towards public speaking and self-reported trait social anxiety, participants were asked to state their agreement, on 8-point likert scales, on the following sentences: “I like to speak in public” and “I can easily become anxious in social situations” (1- strongly disagree; 8 – strongly agree). These questions and the bFNE were only asked after task completion to avoid inducing task anticipation anxiety or evaluation concerns during baseline physiological recordings. Finally, they rated on 7-point likert scales, their experience during the task according to the dimensions: from relaxing (1) to stressful (7); and from unpleasant (1) to pleasant (7). The questionnaire assessing self-reported social anxiety, attitudes towards public speaking and subjective ratings of participants’ experience during the task was not administered to the first 6 participants (Experimental group: n = 2; Control group: n = 4) due to an experimental error.

### Analyses of psychophysiological data

Non-specific-SCR or spontaneous fluctuations in skin conductance (NS-SCRs) analyses were carried out using the SCRalyze b2.18 software (http://pspm.sourceforge.net/ ^32^) according to a convolution model to estimate the area and the curve (AUC), or skin-conductance level-corrected time integral. The AUC is closely related to the conventional methods of estimation of the number and amplitude of NS-SCRs (for further details see ref. 32). This method has been shown to be a better predictor of autonomic arousal than conventional analysis, and has the advantages of not requiring subjective evaluations as well as being computationally inexpensive^32^. HR was estimated with the HRV function implemented in Labchart (v 8.1). Visual inspection and manual correction was carried out to correct unidentified or misidentified peaks. Data from three participants (control group) were discarded from HR analyses due to technical failure during pulse recording. The entire five-minute periods during rest and speech preparation were analysed to estimate baseline and task NS-SCRs and HR levels, respectively. Skin conductance levels and HR were entered into separate repeated measures ANOVAs with Time (Baseline; Task) and Group (Experimental; Control) as within- and between-subjects factors, respectively.

## Results

### Psychophysiological Data

**Figure 2:**
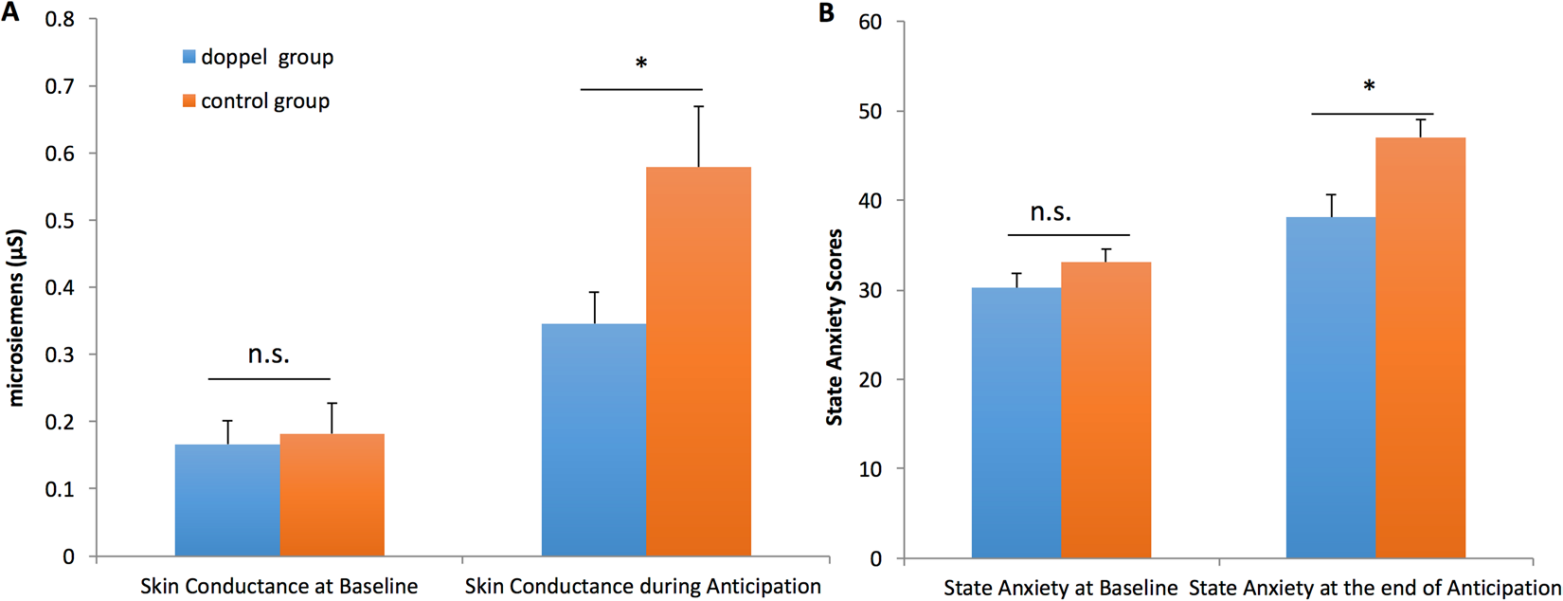
Average Skin Conductance levels across conditions and groups (**A**), and average state anxiety scores (**B**). Error bars indicate S.E.M.

The analysis of NS-SCRs levels revealed a significant main effect of Time (F(1, 50) = 36.69, p < 0.001, ƞ^2^ = 0.42) confirming, as expected, that speech anticipation resulted in an overall increase in physiological arousal (see Fig. 2A), providing a proof that the task was successful in eliciting arousal. The main effect of Group was not significant (F(1, 50) = 3.21, p = 0.079, ƞ^2^ = 0.060). Crucially, the critical Time x Group device (F(1, 50) = 5.23, p = 0.027, ƞ^2^ = 0.095) interaction was significant. Follow-up independent t-tests confirmed lower skin conductance levels in the Experimental group than in Control group during the task (t(1, 50) = 2.24, p = 0.029, Hedges’ g = 0.54) but no difference at baseline (t(1, 50) = 0.271, p = 0.79, Hedges’ g = 0.074). We then investigated changes in average Heart Rate. While the main effect of Time (F(1, 47) = 30.02, p < 0.001, ƞ^2^ = 0.39) on HR was significant because HR increased during anticipation relative to baseline, the Time x Group interaction (F(1, 47) = 0.47, p = 0.50, ƞ^2^ = 0.010) was not significantly different. Furthermore, the main effect of Group was not significant (F(1, 47) = 0.67, p = 0.42, ƞ^2^ = 0.014).

### Subjective measures

To assess differences in self-reported anxiety at baseline and after speech preparation, pre and post STAI scores (see Table 1) were entered into a repeated measures ANOVA with Time (baseline and post-task) and Group (Experimental; Control) as within- and between-subjects factors, respectively. Results confirmed that speech preparation successfully induced anxiety in the participants, as revealed by the main effect of Time (F(1, 50) = 53.91, p < 0.001, ƞ^2^ = 0.52). The interaction Time x Group (F(1, 50) = 4.14, p = 0.047, ƞ^2^ = 0.077, see Fig. 2B) and the main effect of Group (F(1, 50) = 6.75, p = 0.012, ƞ^2^ = 0.12) were also significant. Independent t-tests revealed no group differences in anxiety at baseline (t(1, 50) = 1.34, p = 0.19, Hedges’ g = 0.36) but lower levels of anxiety in the Experimental group after speech preparation (t(1, 50) = 2.79, p = 0.007, Hedges’ g = 0.77). We also carried out independent t-tests on the participants’ ratings on the task experience as relaxing/stressful and unpleasant/pleasant. Results showed that participants in the Experimental group rated the experience as less stressful than those in the control group (t(1, 44) = 2.35, p = 0.023, Hedges’ g = 0.68). Ratings on the pleasantness question did not reach statistical significance (t(1, 44) = 1.94, p = 0.059, Hedges’ g = 0.56). Control analyses revealed no differences between groups on the obtained trait measures: (i) fear of negative evaluation (t(1, 50) = 0.30, p = 0.76, Hedges’ g = 0.082); (ii) social anxiety (t(1, 44) = 0.15, p = 0.88, Hedges’ g = 0.044); (iii) and attitude towards public speaking (t(1, 44) = 0.43, p = 0.67, Hedges’ g = 0.12).

**Table 1:** Average (s.d.) scores on each questionnaire in the Experimental and Control groups. *p < 0.05.

	STAI pre	STAI Post *	bFNE	Social anxiety	Public speaking	Pleasantness	Stressfulness*
Experimental Group	30.2 (8.0)	38.1 (12.6)	36.1 (9.8)	5.00 (2.2)	3.90 (2.2)	5.36 (1.2)	2.96 (1.5)
Control Group	33.1 (7.5)	47.0 (10.3)	37.0 (10.2)	5.09 (1.8)	3.65 (1.9)	4.39 (1.8)	4.09 (1.7)

## Discussion

We tested the efficacy of a new wearable device on calmness using a task that typically induces high anxiety, namely, the preparation and anticipation of giving a short public speech to an unfamiliar audience. *doppel* gives an on-demand, discrete, user-controlled, heartbeat-like vibration applied through a wristband and it aims at modulating the alertness or calmness of the user by adjusting the frequency of the “tactile heartbeat”. In the present experiment, we hypothesized that a slow heart-rate, as opposed to the absence of any vibro-tactile stimulation, would enable participants to remain calmer during the anticipation of public speech. To that end, we told participants that *doppel* was a blood pressure monitoring device, and only for one of the two groups we turned on *doppel* to deliver its tactile vibrations during the anticipation of public speech. Additionally, subsequent oral debriefing confirmed that participants believed that they would actually give the speech and thought that, indeed, *doppel* monitored blood pressure.

Even though, at the beginning of the experiment, the two groups displayed comparable levels of arousal (as measured by Skin Conductance) and of state anxiety, participants in the *doppel* active condition showed a significantly smaller increase in arousal compared to the control group. Similarly, at the end of their speech preparation, and before they were debriefed, participants in the *doppel* active condition reported a significantly smaller increase in their state anxiety compared to the control group. No significant differences in average heart rate were found as a result of *doppel* use. Finally, participants in the *doppel* active condition found the task significantly less stressful than participants in the control condition. Taken together, the results highlight that the use of *doppel* had a clear and significant calming effect in both physiological measures of arousal and subjective reports of anxiety during a task that is effective in inducing social stress, suggesting that *doppel* enabled participants to stay calmer and less anxious, as compared with the condition where the device was worn but was not performing its intended function.

Previous studies have used pharmacological means to study anxiolytic-like effects during anticipation of public speech or during simulated public speaking^33^. For example, de Oliveira and colleagues^33^ used intranasal administration of oxytocin, a neuropeptide that is known to be involved in anxiety, as well as cardiovascular and hormonal regulation, and showed that the oxytocin, but not the placebo, group had lower skin-conductance levels during anticipation and actual speech. Biofeedback techniques have also been used to down-regulate anxiety and physiological reactions during emotional situations^27, 28^. In particular, autonomic regulation training through biofeedback has been shown to reduce subjective and physiological arousal during public speaking^27^. Here, we add to this literature by showing that the use of heartbeat-like vibrotactile stimulation, as opposed to its absence, can also have an anxiolytic effect. These two techniques (i.e. biofeedback and *doppel* use), however, differ considerably in terms of the expectations and attention given to the exteroceptive stimuli. Unlike in biofeedback or false feedback approaches, in our active *doppel* condition, participants were not instructed to regulate their heart rate as a function of these vibrations. Rather than feedback, doppel stimulation is thought to be related to physiological entrainment mechanisms that are not dependent on direct attention or appraisals of interoceptive-exteroceptive matching.

Numerous past studies have also shown how our bodies, including physiological cycles such as the respiratory and cardiac cycles, entrain to different rhythms and how such responses may influence mood^11–14, 34^. Reviewing a large body of relevant research, Phillips-Silver and colleagues^35^ show that the ability to perceive and synchronize to a beat is possible across different sensory modalities. The authors suggest a general model of entrainment that builds upon pre-existing adaptations that allow us to perceive and produce rhythmic stimuli, as well as integrate their production and perception with sensory feedback. Extending the effects of rhythms on mood, beyond entrainment per se, Tajadura-Jiménez, Väljamäe and Västfjäll^36^ reported that heartbeat sounds and their rhythm significantly affected participants’ physiological reactions as well as the emotional judgments of pictures, and their recall. In that study, listening to heartbeat sounds resulted in a small but significant increase in participants’ heart rate with respect to the silence condition, and the sounds significantly influenced emotional responses to affective visual stimuli. In particular, a fast-rate heartbeat (e.g. 110 bpm) sound resulted in higher arousal ratings and enhanced pictures’ memorability during a free-recall task. It is noteworthy that in the present study we did not find any evidence of significant modulations of heart rate, other than the overall increase of heart rate during the anticipation period. This is not surprising given that typically entrainment effects on heart rate are relatively small^11, 12, 36^, and in the present study we employed a task (i.e., anticipation of public speaking) that is meant to increase heart rate. Entrainment effects on physiological activity have been mostly studied in response to musical stimuli^11–14^. However, tempo-related entrainment effects on heart rate are less consistent than in other physiological indices, such as skin conductance and respiration frequency, especially when entraining to slow tempo stimuli^11, 12, 37, 38^. It is possible that this might be related to the non-linear dynamics of cardiac responses to stressors or emotional stimuli. The continuous fluctuations of heart rate due to excitatory and inhibitory regulation processes may explain why average heart rate, like the one reported here, is a less sensitive index to entrainment effects.

Unlike past studies that often use auditory feedback of heartbeats^29, 36, 39, 40^, instead of sounds, we used heartbeat-like tactile stimulation to examine the effects of a calming “heartbeat” rate on anxiety. While no significant differences were observed in participants’ heartrate, the consistent patterns of changes in skin conductance levels and subjective anxiety reports support the hypothesis that the presence of a slow rhythm, which in the present study was instantiated as an auxiliary slow heartbeat delivered through *doppel*, can influence physiological arousal and mood. We used slower frequencies from the participants’ own heart rate because, as previous research has suggested, slower rhythms tend to be associated with low arousal and subjective states of positive or calm emotional states^12, 18^. However, it remains to be tested whether faster or equivalent frequencies to the participant’s heart rate can have similar effects. It is possible that anxiolytic effects can be observed to a range of heart-like vibrotactile stimulation frequencies and even that the “ideal” frequency varies from person to person. Importantly, while we here show the calming effects for slower frequencies, *doppel* users can select the vibrating frequency that corresponds better with their state and possible the one that seems to be more efficient for their own needs and lifestyles.

While the precise underlying neurophysiological mechanism by which *doppel* elicits such effects was not directly investigated in the present study, we suggest that the calming effect may be mediated by neural responses to the rhythmic tactile input that the device delivers, especially neural responses in areas that are involved in somatosensation and interoception such as the somatosensory cortex and the insula^41,42^ and in emotion and rhythm processing such as the basal ganglia^43^. Future studies must specifically investigate such neural responses in parallel with the attenuated changes in skin conductance to complement the significant and converging physiological and subjective measures of reduced arousal and anxiety obtained through the use of *doppel*, as reported here.
